# Effect of a multifaceted educational program for care staff concerning fecal incontinence in nursing home patients: study protocol of a cluster randomized controlled trial

**DOI:** 10.1186/s13063-015-0595-3

**Published:** 2015-03-01

**Authors:** Lene Elisabeth Blekken, Anne Guttormsen Vinsnes, Kari Hanne Gjeilo, Siv Mørkved, Øyvind Salvesen, Christine Norton, Sigrid Nakrem

**Affiliations:** Faculty of Nursing, Sør-Trøndelag University College (HiST), Postbox 2320, 7004 Trondheim, Norway; Department of Cardiothoracic Surgery, Department of Cardiology and National Competence Centre for Complex Symptom Disorders, St. Olavs Hospital, Trondheim University Hospital, Postbox 3250, 7006 Trondheim, Norway; Department of Circulation and Medical Imaging, Norwegian University of Science and Technology (NTNU), 7491 Trondheim, Norway; Department of Public Health and General Practice, Norwegian University of Science and Technology (NTNU), 7491 Trondheim, Norway; Department of Cancer Research and molecular Medicine, Norwegian University of Science and Technology (NTNU), 7491 Trondheim, Norway; Faculty of Nursing and Midwifery, King’ College London, 57 Waterloo Road, London, SE1 8WA UK; Clinical Service, St Olavs Hospital, Trondheim University Hospital, Postbox 3250, 7006 Trondheim, Norway

**Keywords:** Fecal incontinence, Nursing homes, Long-term care, Old patients, Care processes, Nursing, Protocol, Cluster-randomized trial

## Abstract

**Background:**

Fecal incontinence has a high prevalence in the older population, which cannot be explained by comorbidity or the anatomical or psychological changes of aging alone. Fecal incontinence leads to a high economic burden to the healthcare system and is an important cause of institutionalization. In addition, fecal incontinence is associated with shame, social isolation and reduced quality of life. The importance of identifying treatable causes in the frail elderly is strongly emphasized. It is recommended that an assessment of fecal incontinence should be implemented as part of an evaluation of older patients. Although there is a substantial evidence base to guide choice of implementation activities targeting healthcare professionals, little implementation research has focused on the care of older people nor involved care processes or care personnel. This study is based on the assumption that fecal incontinence among nursing home patients can be prevented, cured or ameliorated by offering care staff knowledge of best practice through a multifaceted educational program. The primary objective is to test the hypothesis that a multifaceted educational program for nursing home care staff on assessment and treatment of fecal incontinence reduces patients’ frequency of fecal incontinence.

**Methods/design:**

The study is a two-armed, parallel cluster-randomized controlled trial. Primary outcome is the frequency of fecal incontinence among patients. Sample size calculations resulted in a need for a total sample of 240 patients. Twenty nursing home units in one city in Norway will be recruited and allocated to intervention or control by an independent statistician using computer-generated tables. The intervention is a multifaceted educational program. Units in the control arm will provide care as usual. The intervention period is 3 months. Data will be collected at baseline, 3, and 6 months. Data will be analyzed using mixed effect models with the cluster treated as a random effect.

**Discussion:**

This study is the first randomized controlled trial specifically focusing on this neglected area. The result of the study will give evidence for best practice for continence care in nursing homes, and organizational advice concerning implementation strategies.

**Trial registration:**

ClinicalTrials.gov: NCT02183740, registered June 2014.

## Background

Fecal incontinence (FI) is defined by the International Consultation on Incontinence as ‘the involuntary loss of liquid or solid stool that is a social or hygienic problem’ [1]. FI has a higher prevalence in the elderly population than in younger people, which cannot be explained by comorbidity or the anatomical and psychological changes of aging alone [2]. In the nursing home (NH) population, previous studies suggest a prevalence of FI between 10 and 69% [3-5], but it is most often reported to be between 40 and 55% [1,5,6]. The varying prevalence may be due to the lack of a consistent definition of FI, although differences in the quality of continence care in the NHs might also be an explanation [6,7].

FI leads to a high direct and indirect economic burden to the healthcare system, and is an important cause of institutionalization of older patients [1,2,7]. In addition, FI is associated with shame, social isolation and reduced quality of life [1,8,9]. FI among older patients has a more complex etiology compared to the younger population [2,6], and the importance of identifying treatable causes of FI in frail older people, rather than just managing symptoms passively, is strongly emphasized [1]. The level of awareness among health personnel regarding appropriate assessment and treatment options for FI seems limited [1,10-12]. Further, there are indications that both older patients themselves and health personnel consider FI to be a normal part of aging for which nothing can be done [9,11]. It is recommended that an assessment of FI should be implemented as part of an evaluation of older patients [1,2,13].

There is a substantial evidence base to guide choice of implementation activities targeting healthcare professionals in general [14-17]. However, relatively little of the implementation research has focused on the care of older people or involved care processes or care personnel [18]. Even though the evidence is not fully conclusive, implementation research suggests that the most effective method for changing the behavior of health personnel in long-term care settings involves multifaceted educational efforts such as written materials or toolkits combined with individual educational visits, small group training or feedback [14,17,19].

This study is based on the hypothesis that FI among NH patients can be prevented, cured or ameliorated by offering NH care staff knowledge of best practice through a multifaceted educational program on assessment and treatment of FI. The study has been developed according to guidelines for developing and testing complex interventions [20-23]. As there are very few trials either on the treatment of FI in NH patients or on continence education programs for care staff, it was considered necessary to investigate feasibility before evaluating the complex intervention with a randomized controlled trial [20]. Thus, a pilot study was conducted in autumn 2013. The results are not yet published, but the experiences and results have been used in the planning of this cluster-randomized controlled trial.

### Aims and objectives

The overall aim of this study is to achieve a reduction in bowel leakage and accidents for NH patients by altering the quality of continence care among registered nurses (RNs), authorized social educators (ASEs, see below), and care staff in general. The primary objective is to test the hypothesis that a multifaceted educational program for NH care staff on assessment and treatment of FI reduces patients’ frequency of FI.

Secondarily, the trial will investigate the effect of a multifaceted educational program for NH care staff on 1) remission of FI among patients with FI present at baseline or the incidence of new cases of FI among patients identified as continent at baseline; 2) change in NH patients’ FI-related concerns such as mood, constipation, diarrhea and skin condition; 3) increased knowledge among RNs and ASEs; 4) change of practice among RNs, ASEs and care staff in general; 5) and reduction in costs related to FI management. The study also intends to investigate correlates of FI in the NH population.

## Methods/design

The study is a parallel two-armed cluster randomized controlled trial (C-RCT) with a repeated cross-sectional design. As there will be considerable overlap between patients included at the different data-collection time points, some outcomes will be treated as if they come from a cohort design.

### Setting

In Norway, the municipalities have a statuary obligation to provide NH care to those who need it. Most Norwegian NHs are owned and run by the municipalities, and financed by a combination of taxes and patient payment. NH size varies between 20 and 120 beds, divided into units most commonly with 15 beds. NHs are managed by RNs and have an agreement with a general practitioner (GP) who visits the NH once a week. There are no legal requirements for staff-to-patient ratios or specifications for qualifications required for workers [24]. However, NHs have RNs and/or ASEs on duty 24 hours a day, and according to unpublished information from Statistics Norway the staff comprises on average 31% RNs/ASEs, 45% licensed practical nurses (care education on high school level most often before age 18) , and 24% healthcare aides (no vocational health education). Statistics Norway has overall responsibility for official statistics in Norway. In Norway, an ASE has a bachelor’s degree and provides daily care to persons in need, particularly those with intellectual disability, including dementia. ASEs have a defined healthcare and pharmacological competence [25].

### Intervention

The educational program has been developed according to recommendations from implementation research, pedagogic theory and experience from members of the project group [14,16,19,26-31]. A research group comprising four researchers will facilitate the educational program and will be trained as a unified team to enhance standardization of the intervention. Educational content, pedagogical methods and a paper-based guideline for nurse-led assessment and treatment of FI (the FI-guideline, see below) were developed for this study by expert consensus and were evaluated in the pilot study.

#### Content of the multifaceted intervention

*The FI-guideline* is based on best practice recommendations [1,13,32,33] and will be introduced to the RNs/ASEs in the intervention NHs during the workshop (see below). The FI-guideline facilitates a systematic assessment of bowel symptom history and bowel patterns. As FI among NH patients is considered to have a complex etiology, the guideline encourages the RN/ASE to consider a range of possible causes. Examples are loose stools, immobility, cognitive impairment, impaction and use of laxatives. Based on this assessment, the RN/ASE defines a nursing diagnosis, for example: ‘FI related to loose stools, possibly due to excessive laxatives, urgency and reduced mobility. This leads to FI episodes with loose stool and red perineal skin’. The guideline then offers a range of possible interventions. The result of the paper-based assessment, the nursing diagnosis and interventions, is then documented in the patient’s EPR as an individualized care plan.

*One one-day educational meeting* (7 hours) is defined by the Cochrane Effective Practice and Organization of Care (EPOC) as ‘participation of healthcare providers in conference, lectures, workshops or traineeships’ [14]. The educational meeting will be organized as an interactive workshop, which targets knowledge, attitudes, and skills. The workshop will be conducted in a local meeting room in each intervention NH. Part one of the workshop includes the RNs/ASEs filling in a knowledge test (which is part of the data collection). However, by organizing it as a part of the workshop, the pedagogical intention is to facilitate learning, as it is possible to find the answers in the following theoretical input. Part two of the workshop is case-based discussion concerning the FI-guideline. As individualization of the nurse’s diagnoses and the interventions is essential, an important pedagogical intention is to empower the RNs/ASEs’ clinical and critical thinking. Another important issue is how to integrate the use of the guideline with the electronic patient record (EPR) system. The topics for the educational meeting, including the FI-guideline, will be made available for the staff as printed educational material [14].

*A local opinion leader* is defined by EPOC as ‘use of providers nominated by their colleges as educationally influential’ [14]. The opinion leader will be recruited after the educational meeting by the informant method [34] by asking the care manager who is considered to be a principle source of influence. One opinion leader per unit will be recruited. The opinion leader will, together with the care manager, participate in an additional 1.5 hour educational meeting regarding the role of the opinion leader and care manager for this study. They will also receive contact information for the researcher for support during the intervention period. The care manager has responsibility for facilitating adherence to the program and the guidelines in cooperation with the opinion leader.

*Educational outreach* is defined by EPOC as ‘use of a trained person who meets with providers in their practice setting to give information with the intent of changing the providers’ practice’ [14]. The researcher will meet with the healthcare personnel in the practice setting six times for 1.5 hours each time during the 3-month intervention period. The opinion leader will make an agreement with the researcher on how to work and what to focus on between meetings. The NH care staff as a whole is the target group for the educational outreach and will be invited to participate in the educational meetings throughout the intervention period. Facilitating and empowering the staff’s critical and clinical thinking is the main pedagogical approach. The pilot study identified a culture of discontinuity among staff in reporting important clinical observations and decisions in the EPR as an essential barrier to change. In addition, even if decisions were reported in the EPR, it was a problem that staff did not check the patients’ EPR for changes in the patients’ care procedures, which resulted in the patients not receiving the correct interventions for his/her condition. Thus, it will be important to facilitate NH unit-specific strategies to ensure continuity in FI care for the individual patient.

### Control group

The control group will not receive any educational program and will continue with usual care. Data on ordinary practice will be gathered as part of the data collection procedure in this study (health information on patients, ordinary practice as documented in the EPR, care for patients’ FI, diarrhea and constipation as documented in The Fecal Incontinence in Nursing Home Patient questionnaire [6]).

### Eligibility criteria

Results from the pilot study confirmed that units in Norwegian NHs are comparable with the definition by Norton and colleagues [35]. NH units with similar care staff/patient ratios on the day shift and GP coverage will be selected. NH units designated with a specialty or with an enhanced care staff/patient ratio will be excluded. RNs and ASEs working half time or more are eligible for participation in the workshop and to be recruited as opinion leaders in the intervention group. RNs/ASEs working less than half time or only night shifts are excluded. All care staff members in the NH will be invited to the educational outreach meetings throughout the intervention period. All long-term care patients (1 month or more) are eligible for inclusion.

### Recruitment

Approval will be obtained from the director for health and social affairs in the municipality. The first and last authors will participate in a meeting where all managers for the NHs in the municipality will be gathered. The project will be presented and NHs invited to participate. NHs accepting the invitation will be eligible for selection. NH units will be enrolled until the target patient sample size is reached.

### Randomization and allocation

**Figure 1:**
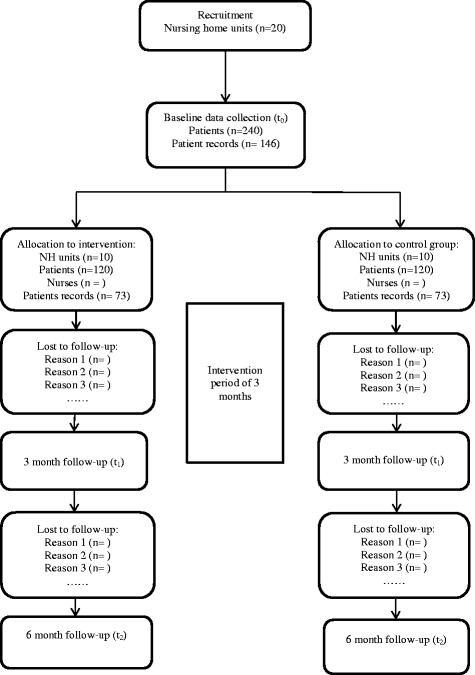
**Trial flow-chart.**

One unit will be defined as one cluster. Two clusters per NH will be recruited. Allocation stages are as follows:

1. NHs will be identified and recruited;
2. Units will be identified and recruited;
3. Patients will be identified;
4. Baseline data collection will take place for units and patients;
5. Allocation will be done by an independent statistician to intervention or control; and
6. RNs/ASEs will be identified and recruited to the intervention (Figure 1).

The clusters will be allocated to the intervention or control arm using minimization [23]. Minimization factors are a) all units from the same NH will be allocated to the same arm and b) cluster size, where NHs will be sequenced in pairs according to size and then randomized to either intervention or control. The randomization method is simple randomization and is computer generated and performed by an independent statistician (Figure 1).

### Outcome measures

The primary outcome measure is frequency of FI among patients 6 months after start of the intervention, as measured by The interRAI Long-Term Care Facilities Assessment System (interRAI LTCF) [36], section H3: Bowel continence with the categories 0 to 5 (0 = continent, 1 = continent with stoma, 2 = seldom incontinent, 3 = occasionally incontinent, 4 = often incontinent, 5 = incontinent). The interRAI LTCF is an internationally validated questionnaire regarding long-term care patients’ health conditions. In order to get some additional information about the severity of FI and urgency, a Norwegian version of the St. Mark’s anal incontinence score [37] will be used. The St. Marks grading system is based on the type and frequency of anal incontinence (gas, fluid, or solid) and the impact on daily life, the need to wear a pad/diaper and/or anal plug, the use of constipating medication and the presence of urgency. It gives a total score from 0 (complete continence) to 24 (complete incontinence).

The study has the following secondary outcome measures:

1. *Remission of FI* among patients identified with FI at baseline, or *incidence* of new cases of FI among patients identified as continent at baseline, measured by interRAI LTCF section H3 and the St Marks score.
2. *Change in FI related concerns* measured by interRAI LTCF: section E: Mood and behavior, section F: Psycho-social wellbeing, section H1: Urinary continence, section J: State of health - Constipation and diarrhea, section L: Skin condition, and section M: Participation in activities.
3. *Change in knowledge* among RNs/ASEs measured by multiple choice tests developed by the researchers according to established guidelines [38].
4. *Change in documented care for FI by health personnel as registered in the EPR*: RNs/ASEs will extract data from the EPR. The instrument N-Catch [39] will be used for this purpose. N-Catch is an audit instrument for nursing reports in the EPR. N-Catch is translated into Norwegian and developed based on the validated D-Catch [40] and Cat-ch-ing [41]. Change in care will also be measured by the Fecal Incontinence in Nursing Home Patients questionnaire [6] where RNs/ASEs are offered a list of interventions for FI, urinary incontinence, diarrhea and constipation and asked to identify what is done for each individual patient.
5. *Change in cost* of FI management measured by mapping the use of products (diapers, pads, plugs) and bowel medications measured by interRAI LTCF section H: Continence - remedies, section N: Medications, and the Fecal Incontinence in Nursing Home Patients questionnaire.
6. *Correlates of FI* among NH patients measured by interRAI LTCF: section C: Cognitive functioning, section D: Communication and vision, section G: Functionality and mobility, section I: Medical diagnoses, section J: Health condition, section K: Mouth- and nutrition status, section N: Medications, and section O: Treatment, examinations/procedures.

Process evaluation will include attendance at the pedagogical program and use of the FI-guideline. Evaluation will be conducted by using checklists administered by the researchers: proportion RNs/ASEs within eligibility criteria participating in the workshop, proportion of staff participating in the educational outreach meetings, proportion of patients assessed by the FI-guideline, and proportion of patient assessments resulting in an individualized care plan in the EPR.

### Background variables

Organizational characteristics of the NHs and background information on patients’ sex, medical status and length of stay in the NH will be obtained. Background variables for the RNs and ASEs are age, sex, educational level, years since registered/authorized and length of employment at the present site.

### Sample size

Sample size calculations are based on the primary outcome: frequency of FI among patients. The power calculations have taken into account the results from the pilot study. The pilot study identified the primary outcome variable to be skewed to the right, and methodological consideration resulted in a dichotomization of the primary outcome variable with a cut off between 2 (continent, continent with a stoma and seldom incontinent) and 3 (occasionally incontinent, often incontinent and incontinent). Based on results from the pilot study, we hypothesized that a reasonable and clinically important effect size in the intervention group compared to the control group would be 15% between the two groups in proportions with FI (score of 3 to 5). As the design is a cluster randomized trial, we need to adjust for clustering. The intracluster correlation coefficient (ICC) is estimated to be 0.04. The estimate is based on published patterns in ICCs [23,42-44], and results from the pilot study where the ICC was calculated to be 0.038. In addition, we had access to the FI variable with the categories 0 to 4 from an epidemiological study of 980 NH patients in Trondheim municipality [6] with an ICC calculated as 0.028. Based on the assumptions of the mixed logistic binominal model, 5% level of significance, test strength of 80%, an average cluster size of 15 patients, and an ICC of 0.04, a study population of 103 patients in each arm of the C-RCT is needed. The number of individuals in each cluster is set because each unit has a fixed number of beds. Assuming a 15% dropout, the sample needed is 120 patients in each arm. This means a total of 240 patients and about 20 NH units (Figure 1).

In addition, the number of patients records needed for data extractions, was calculated. N-Catch measures the quality of the content in the EPR on a scale from 0 to 32 where 0 is low quality and 32 is high quality [39-41]. Based on the assumption of a paired *t*-test, a 5% level of significance, test strength of 80%, an effect size of 3 points and an ICC of 0,04, records from 6 patients per cluster is needed for a total of 146 records (Figure 1).

### Data collection methods

Data are collected at baseline (t_0_), after 3 months (t_1_ = end of intervention), and after 6 months (t_2=_primary time of assessment). A research assistant will, together with the first author, give information and training on completion of the questionnaires and data extraction from EPR at baseline. RNs/ASEs will then be responsible for filling in the questionnaires about the patients’ health condition (proxy) and extracting the data from the EPR. Organized alphabetically by last name on a list, the EPRs of the first six patients per cluster will be extracted. Time scheduled for the information meetings is 2 to 3 hours per NH. The ward manager will fill in a form on organizational characteristics. When completed, the first author and research assistant will collect the data forms. At both t_1_ and t_2_ the research assistant will deliver, give necessary additional information and collect the completed forms. NHs will be offered economic compensation for the data collection.

### Blinding

Baseline measurements will be done before randomization. A research assistant who will be blinded to group allocation will inform, deliver and collect the questionnaires/data after 3 months (t_1_) and 6 months (t_2_)_._

### Statistical analyses

Descriptive statistics will be used to present the population and the characteristics of the three groups (units, RNs/ASEs, and patients). As this is a C-RCT, the model of analysis needs to consider the effect of clustering. Analyses must also allow for inclusion of covariates at both the individual and cluster level. Relevant covariates at the individual level in a multiple logistic regression model are age, sex, length of stay in NH, cognitive performance, mobility, functionality, diarrhea, constipation. Hence, this study will use mixed effect models with the cluster treated as a random effect. Analyses will be computed using Stata 12.1.

#### Primary outcome

As a consequence of the dichotomization of the variable, the primary outcome will be analyzed according to a mixed logistic binominal model. The model will be fitted by maximum likelihood. Because of the relatively high risk of deaths and movement out of clusters, data will be treated as a cross-sectional time series, with the prevalence among all patients present in the cluster at baseline included as a covariate in the analyses.

#### Secondary outcomes

For remission of FI a cohort approach to data analyses with repeated measures with only those identified with FI at baseline and still present at 3 and 6 month follow-up will be included and analyzed according to a mixed logistic binominal model. For incidence of FI a cohort approach to data analyses with repeated measures with only those identified as continent at baseline and still present at 3 and 6 month follow-up will be included and analyzed according to mixed logistic binominal model. For change in FI-related concerns, change in knowledge among RNs/ASEs, change in care, and change in cost the outcomes, dependent on whether they are continuous, ordered or binary, will be treated as cross-sectional time-series and analyzed according to mixed effects models. Correlates of FI will, dependent on whether they are continuous, ordered or binary, be analyzed according to mixed effect models.

### Ethics

The study was approved by the Regional Committee for Medical and Health Research Ethics (REK) (2013/1802/REK North) and by The Norwegian Social Science Data Services (36482/2/MB). NH leaders will be informed and give permission to perform the study in the individual NH. Informed consent will be obtained from RNs/ASEs concerning the knowledge test. An essential ethical consideration in this study is whether or not informed consent should be obtained from patients or their representatives. After evaluating the overall project, the REK authorized RNs/ASEs with dispensations from the duty of confidentiality to gather relevant patient health information in order to measure effect of the educational intervention. Since dispensation was given, consent will not be obtained. The justifications of the conclusion are 1) the process of assessing the patients’ cognitive ability to read and understand information, and the distribution of the information letter to the patients or their representatives, is considered as inconvenient for the patients and time consuming for care staff who would need to undertake this; 2) the gathering of patient data will not involve interviewing or examining patients, and the data in question is based on assessments made by RNs/ASEs who have good knowledge of the patients; and 3) the purpose of the study is to evaluate effect of an educational program for care staff. Patients are not the ones recruited to participate in the intervention. All patient information will be de-identified by care staff before transfer to the researcher. The codebook will be stored separate from the patient data according to storing routines by the responsible research institution, Sør-Trøndelag University College. The study will be performed in concordance with the Helsinki Declaration. The project is registered in the clinical trial registry (NCT02183740).

## Discussion

The aim of this study is to achieve reduction in bowel leakage and accidents for NH patients. The primary objective is to test the hypothesis that a multifaceted educational program for NH care staff on assessment and treatment of FI, reduces patients’ frequency of FI.

Major strengths of this study include thorough investigation of both what is considered best practice for assessment, care and treatment of FI among NH patients and what are considered to be the most effective implementation strategies. The study has a rigorous design with randomization, control and blinding where possible. The intervention is classified as a complex intervention, and the study has been designed according to published recommendations [20,21], where a thorough planning phase included an evaluation of the fit of the different components with a pilot study. In addition, a strength is that we will collect comprehensive information at three levels: NH units, RNs/ASEs and patients. It is of special interest that the educational intervention integrates the FI-guideline of best practice into the EPR as a mean to communicate the assessment and care plan to the staff as a whole. Because of this, we will have the opportunity to evaluate change of practice by investigating the EPR together with the use of the FI-guideline and patient’s health information.

A weakness of the study is the complexity of the intervention with limited possibility to evaluate which of the components in the educational intervention is effective. The more complex the intervention, the harder it is to measure effect [14,20]. With an educational intervention, we also have the problem with the pedagogical ideal versus ideals for an RCT. An important pedagogic ideal is to individualize and adjust pedagogical methods according to the needs of the actual person/group in front of you [15,29-31]. On the other hand, an important ideal of an RCT is that the intervention is as similar as possible for all the participants [45]. In this study, we have agreed that some components will be the same, and some are allowed to vary. For instance, the format of the workshop will be the same, (total hours and themes to be covered), while empowerment strategies, guidance and timeframes for individual themes during the day may vary. During educational outreach, all participants will receive the same number of visits within the same time frame and main themes to be covered, whereas the when and how will vary.

As it is the RNs/ASEs who will fill in the questionnaires on the patients’ health status, there is a risk for proxy bias. To counter for that, both the interRAI manual and the information meetings focus on how to include the patient when possible. However, since about 80% of the NH patients have some kind of cognitive impairment [46], the RNs/ASEs’ clinical judgment of the patients’ health status will be the main source of information. The RNs/ASEs involved in the data collection will also be part of the intervention, which means that they will not be blinded to care interventions. The RNs/ASEs will be informed about the importance of objectivity of observations and assessments at all data collection time points. There is also a risk for detection bias as those who have received education might recognize FI more frequently than before intervention.

This study is the first RCT specifically focusing on this neglected area. The results of the study will give evidence for best practice for FI care in NHs, and organizational advice concerning implementation strategies.

## Trial status

Enrollment for the trial began in April 2014. Recruitment is still in progress. Data collection will continue until approximately June 2015.

## Abbreviations

ASE: Authorized social educator
C-RCT: Cluster-randomized controlled trial
EPOC: Cochrane effective practice and organization of care
EPR: Electronic patient record
FI: Fecal incontinence
GP: General practitioner
ICC: Intra-cluster correlation coefficient
interRAI LTCF: The interRAI Long-Term Care Facilities Assessment System
NH: Nursing home
RN: Registered nurse
